# *Leishmania* spp. genetic factors associated with cutaneous leishmaniasis antimony pentavalent drug resistance: a systematic review

**DOI:** 10.1590/0074-02760230240

**Published:** 2024-09-02

**Authors:** Raphaela Lisboa Andrade Nery, Thaline Mabel Sousa Santos, Luana Leandro Gois, Aldina Barral, Ricardo Khouri, Caroline Alves Feitosa, Luciane Amorim Santos

**Affiliations:** 1 Fundação Oswaldo Cruz-Fiocruz, Instituto Gonçalo Moniz, Salvador, BA, Brasil; 2 Universidade Federal da Bahia, Faculdade de Medicina, Programa de Pós-Graduação em Ciências da Saúde, Salvador, BA, Brasil; 3 Escola Bahiana de Medicina e Saúde Pública, Salvador, BA, Brasil; 4 Universidade Federal da Bahia, Instituto de Ciências da Saúde, Departamento de Ciências da Biointeração, Salvador, BA, Brasil

**Keywords:** *Leishmania* spp, drug resistance, drug susceptibility, cutaneous leishmaniasis, genetic factors, treatment outcome, systematic review

## Abstract

**BACKGROUND:**

Leishmaniasis is a neglected zoonosis caused by parasites of *Leishmania* spp. The main drug used to treat cutaneous leishmaniasis (CL) is the antimoniate of meglumine. This drug, which has strong adverse and toxic effects, is usually administered intravenously, further complicating the difficult treatment. Factors such as *Leishmania* gene expression and genomic mutations appear to play a role in the development of drug resistance.

**OBJECTIVES:**

This systematic review summarises the results of the literature evaluating parasite genetic markers possibly associated with resistance to pentavalent antimony in CL.

**METHODS:**

This study followed PRISMA guidelines and included articles from PubMed, SciELO, and LILACS databases. Inclusion criteria were studies that (i) investigated mutations in the genome and/or changes in gene expression of *Leishmania* associated with treatment resistance; (ii) used antimony drugs in the therapy of CL; (iii) used naturally resistant strains isolated from patients. The Joanna Briggs Institute Critical Appraisal Checklist was used to assess article quality and risk of bias.

**FINDINGS:**

A total of 23 articles were selected, of which 18 investigated gene expression and nine genomic mutations. Of these 23 articles, four examined gene expression and genomic mutations in the same samples. Regarding gene expression, genes from the ABC transporter protein family, *AQP1*, *MRPA*, *TDR1* and *TRYR* were most frequently associated with drug resistance. In one of the articles in which mutations were investigated, a mutation was found in *HSP70* (*T579A*) and in three articles mutations were found in *AQP1* (*A516C*, *G562A* and *G700A*). A limitation of this review is that in most of the included studies, parasites were isolated from cultured lesion samples and drug resistance was assessed using *in vitro* drug susceptibility testing. These approaches may not be ideal for accurate genetic evaluation and detection of treatment failure.

**MAIN CONCLUSIONS:**

The development of further studies to evaluate the genetic resistance factors of *Leishmania* spp. is necessary to elucidate the mechanisms of the parasite and improve patient treatment and infection control.

Leishmaniasis is a zoonotic, neglected disease caused by parasites of the genus *Leishmania (L.)* spp.^1^ More than 431 million people live in endemic areas and are at risk of developing associated diseases, 12 million cases are registered and 2 million new cases occur each year.^2^ ^,^ ^3^ Leishmaniasis occurs in many areas of the world, particularly in developing countries in Central and South America, Southern Europe, the Middle East, the Indian subcontinent and North and East Africa.^4^ Leishmaniasis is one of the most common causes of death from infectious diseases.^5^

This parasite has several species and can cause different clinical manifestations in human infection such as cutaneous leishmaniasis (CL), mucocutaneous leishmaniasis (MCL) and disseminated leishmaniasis (CDL), in addition to the forms of diffuse and visceral leishmaniasis (VL). The most common clinical form is CL, which is characterised by erythematous papules that may develop into roundish ulcers with raised margins, have a limited course and may heal spontaneously.^1^ ^,^ ^6^ *Leishmania* species associated with the development of CL in the New World include *L. braziliensis*, *L. guyanensis*, *L. amazonensis*, *L. panamensis* and *L. aethiopica*, while in the Old World it is *L. tropica* and *L. major*.^3^ CL has been described in southern Europe, Asia, Africa and Latin America.^7^ MCL typically affects the oral, nasal and pharyngeal mucosa, resulting in disfiguring lesions. The disseminated form has been described in Mexico (Northeast) and the United States (Texas),^7^ while MCL occurs in Latin America and Africa, although the latter is less common.^6^ VL is classified as the most severe form as it affects internal organs and can lead to death if left untreated.^7^ ^,^ ^8^

According to the World Health Organization (WHO), Meglumine antimoniate and Sodium stibogluconate (pentavalent antimonial) are the drugs of first choice in most parts of the world, as their overall cure rate is over 90%.^1^ However, various alternative drugs can also be used to treat this disease.^4^ Alternatives to antimonials, which can be administered intravenously or intramuscularly, include miltefosine (administered orally), paromomicin (administered intramuscularly, orally and topically) and pentamidine (administered intravenously or intramuscularly).^5^ ^,^ ^6^

The choice of therapy depends on age, gestation, the presence of comorbidities and the cost-benefit ratio of the drug toxicity used, as side effects may occur.^7^ These drugs are chemotherapeutic agents that are known to have adverse and toxic effects, including hypotension, vomiting, nausea and hallucinations. Additionally, serious adverse effects such as nephrotoxicity and pancreatitis may occur, and in some cases, cardiotoxicity is also a concern. They also need to be administered intravenously, so patients from rural areas must travel long distances to receive the drug, making it very difficult to treat patients comprehensively.^8^

The first evidence of treatment failure due to drug resistance was described in India, north of Bihar, in 1997.^9^ Resistance to *Leishmania* was mainly studied with Antimonial Pentavalent.^10^ Little information is available on the mechanisms of drug resistance in CL, but host, parasite or vector factors play an important role.^11^ Studies have shown that genetic mutations of the parasite and variations in gene expression may contribute to treatment failure.^12^ The flexibility of the *Leishmania* genome is crucial for drug resistance, as the parasite adapts its genetic expression of drug targets and transporters after drug exposure.^3^ In addition, there is evidence that *Leishmania* can influence the genetic expression of proteins involved in drug transport and metabolism in the host cell. Therefore, understanding the genetic modulation of *Leishmania* and its association with drug resistance is crucial to improve treatment efficacy and identify alternative therapeutic targets with lower resistance potential.^13^

Several genetic mechanisms that may lead to drug resistance include gene copy number variations, aneuploidy, small and specific intrachromosomal regions, the presence of extrachromosomal DNA that could be exchanged between strains, and genomic mutations (single nucleotide variations, insertions or deletions).^14^ ^,^ ^15^ In *Leishmania*, variations in gene dosage or chromosome copy number also influence drug susceptibility. In addition, single nucleotide polymorphisms (SNPs) in the drug targets or transporters can lead to drug resistance without change in the gene expression.^14^ Drug resistance in *Leishmania* is complex and few studies have investigated these adaptive mechanisms.^13^ Therefore, it is necessary to identify the possible *Leishmania* genes that may be associated with drug resistance, such as genomic mutations or changes in gene expression. In addition to identifying these genes, it is also important to find a marker that predicts the outcome of leishmaniasis treatment in clinical use. Identification of markers can help in medical treatment by avoiding treatment of the patient with chemotherapeutic agents to which *Leishmania* species may be resistant. Therefore, the aim of this systematic review is to summarise the results of the literature on genetic factors that may be associated with resistance to antimony pentavalent drugs in CL.

## MATERIALS AND METHODS

*Search strategy* - This study is a systematic review following the Preferred Reporting Items for Systematic Reviews and Meta-Analyses (PRISMA) protocol [Supplementary data (Tables I-II)],^16^ addressing the genetic factors associated with antimonial drug resistance in CL.

The database searches were performed on February 11, 2023 using PubMed (National Centre for Biotechnology Information NCBI), SciELO (Scientific Electronic Library Online) and LILACS (Literatura Latino-Americana e do Caribe em Ciências da Saúde). The keywords were identified using Medical Subject Headings (MeSH) to construct the following search algorithm: (“cutaneous leishmaniasis” OR “*Leishmania braziliensis*” OR “*Leishmania amazonensis*” OR “*Leishmania guyanensis*” OR “*Leishmania major*” OR “*Leishmania viannia*”) AND (“drug resistance” OR “treatment failure” OR resistance OR “drug susceptibility” OR “treatment outcome”) AND (mutation* OR gene* OR polymorphism OR SNP OR expression OR RNA-seq).

*Eligibility criteria* - The following inclusion criteria were used to select eligible articles to be included in this study: (i) articles investigating mutations in the genome and/or changes in gene expression of *Leishmania* associated with therapy resistance; (ii) articles that used antimonial drug in the therapy of CL; (iii) studies that used naturally resistant strains isolated from patients; (iv) articles in English, Portuguese or Spanish; (v) articles published from the year 2000 onwards.

The following exclusion criteria were also applied in the selection of articles: (i) review articles or case reports; (ii) articles in which the presence of *Leishmania* genetic factors in CL was not measured, quantified or identified; (iii) articles in which only animal studies were reported; (iv) articles in which visceral or diffuse leishmaniasis was studied; (v) studies in which laboratory-induced mutations or expressions were used; (vi) samples from patients with chronic infectious diseases; (vii) articles in which drugs other than antimony were used.

*Data extraction* - Initially, two authors (RLAN and TMSS) independently read the titles and abstracts using the selection criteria for the first selection. For the second selection, the full articles were read and reviewed for eligibility. Disagreements between the two reviewers were analysed by a third reviewer (LAS).

The following data was then extracted from the selected articles: type of study, title, authors, year of publication, location of study, type of sample, *Leishmania* species, classification of resistance, method used to identify mutations, genes analysed with genomic mutation, method used for gene expression analysis, gene expression analysed, genes associated with resistance, genes associated with susceptibility. The data obtained were summarised and synthesised in a Microsoft Excel spreadsheet and the results were presented in frequencies.

The “Joanna Briggs Institute Critical Appraisal Checklist for Analytical Cross-Sectional and Cohort Studies” was used to assess the quality of the articles and the risk of bias. To assess the risk of bias, the percentage of “yes” answers on the checklist was calculated for each article. Articles were classified as low risk of bias at 70% or more, medium risk of bias at 50-69%, and high risk of bias at 50% or less.^17^

*Registration* - The protocol of this systematic review was registered in International prospective register of systematic reviews (PROSPERO) by registration number: CRD42021225134. This protocol proposed to investigate genetic factors associated with resistance to different drugs used to treat leishmaniasis, such as amphotericin B, antimonials and miltefosine, but the search only found articles evaluating antimonials and the results were presented in this review.

## RESULTS

A total of 1,368 articles were found on the PubMed (1,312), SciELO (7) and LILACS (49) platforms. Inclusion and exclusion criteria were applied and a total of 23 articles were selected for this study (Figure). All cases investigated in the included articles were CL. All included articles included presented a low risk of bias according to the Joanna Briggs Institute Critical Appraisal Checklist [Supplementary data (Table I)].

**Figure:**
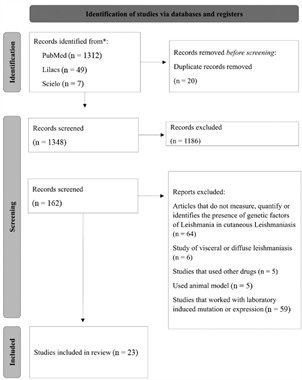
Flowchart of this systematic review study selection.

Of the 23 articles selected, thirteen (56.5%) were cross-sectional studies and ten (43.5%) were cohort studies. In more than half of the studies, the samples were from Iran (60.9%), while the other studies were from Central and South America (34.8%) and did not provide information on the place of origin of the samples (4.3%). The Central and South American countries included were Brazil (21.7%), Peru (8.7%) and Panama (4.3%), as well as one study with samples from Suriname (4.3%), which also used samples from Brazil and Peru (Table I). Among the included articles, there is no consensus on the classification of cure and treatment failure. Four articles (17.4%) defined treatment failure after three or more cycles of the drug without cure, six studies (26.1%) considered three months of treatment without cure, four articles (17.4%) considered no cure after one cycle, one study (4,3%) considered active damage after two cycles, one study (4.3%) considered relapse after six months of treatment, five studies (21.8%) considered EC50 (half maximum effective concentration), and two studies (8.7%) provided no information (Tables II-III).

**TABLE I:** Characteristics of the included articles (n = 23)

Author and year	Type of study	Sample location	Sample type	Sample size	*Leishmania* specie	Assessment of genetic factors	Score Joanna Briggs
Torres, 2010^(31)^	Cross-sectional	Brazil	Parasite obtained from the lesion	TF = 13 TR = 24	*L. braziliensis* *L. guyanensis*	Expression	100%
Adaui V, 2011^(33)^	Cross-sectional	Peru	Parasite obtained from the lesion	TF = 10 TR = 11	*L. braziliensis*	Expression	100%
Adaui V, Maes, 2011^(54)^	Cohort	Peru/ Brazil/ Surinam	Parasite obtained from the lesion	TF = 11 TR = 15 ND = 3	*L. braziliensis*	Mutation	90.9%
Alizadeh R, 2011^55^	Cross-sectional	Iran	Parasite obtained from the lesion	TF = UN TR = UN 97	*L. tropica* *L. major*	Mutation	100%
Kazemi-Rad E, 2013a^(47)^	Cross-sectional	Iran	Parasite obtained from the lesion	TF = 1 TR = 1	*L. tropica*	Expression	100%
Kazemi-Rad E, 2013b*^56^	Cross-sectional	Iran	Parasite obtained from the lesion	TF = 1 TR = 1	*L. tropica*	Expression	100%
Torres DC, 2013^(12)^	Cohort	Brazil	Parasite obtained from the lesion	TF = 19 TR = 29	*L. braziliensis* *L. guyanensis*	Mutation	90.9%
Eslami G, 2016^57^	Cohort	Iran	Biopsy of injury	TF = 4 TR = 12	*L. major*	Expression	100%
Hajjaran H, 2016^58^	Cross-sectional	Iran	Parasite obtained from the lesion	TF = 14 TR = 18	*L.tropica*	Expression	100%
Ghobakhloo N, 2016^59^	Cohort	Iran	Parasite obtained from the lesion	TF = 5 TR = 5	*L. major*	Expression	90.9%
Barrera MC, 2017^(13)^	Cross-sectional	UN	Parasite obtained from the lesion	TF = 10 TR = 9	*L. panamensis*	Expression	87.5%
Oliaee RT, 2018^(29)^	Cohort	Iran	Parasite obtained from the lesion	TF = 14 TR = 14	*L. tropica*	Expression and mutation	90.9%
Rugani JN, 2019^(18)^	Cross-sectional	Brazil	Biopsy of injury	TF = 2 TR = 1	*L. braziliensis*	Expression and mutation	100%
Mohebali M, 2019^(43)^	Cross-sectional	Iran	Parasite obtained from the lesion	TF = 7 TR = 7	*L.tropica*	Expression	100%
Alijani Y, 2019^(19)^	Cross-sectional	Iran	Biopsy of injury	TF = 5 TR = 145	*L. major*	Expression and mutation	100%
Ahmadian S, 2019^60^	Cohort	Iran	Parasite obtained from the lesion	TF = 9 TR = 9	*L. major*	Expression	90.9%
Restrepo CM, 2019^(46)^	Cross-sectional	Panama	Parasite obtained from the lesion	TF = 1 TR = 2	*L. panamensis*	Mutation	100%
Fozongari F, 2020^61^	Cross-sectional	Iran	Biopsy of injury	TF = 15 TR = 15	*L. tropica*	Mutation	100%
Eslami G, 2021^62^	Cross-sectional	Iran	Biopsy of injury	TF = 5 TR = 3	*L. major*	Expression	100%
Somee R, 2021^(20)^	Cross-sectional	Iran	Biopsy of injury	TF = 2 TR = 5	*L. major*	Expression and mutation	100%
Zabala-Pe**ñ**afiel, 2021^(36)^	Cross-sectional	Brazil	Parasite obtained from the lesion	TF = 7 TR = 5	*L. braziliensis*	Expression	100%
Nourbakhsh, 2021^(34)^	Cross-sectional	Iran	Biopsy of injury	TF = 5 TR = 3	*L. major*	Expression	100%
Bahrami, 2022^63^	Cross-sectional	Iran	Biopsy of injury	TF = 10 TR = 15	*L. tropica*	Expression	100%

*RNAseq; UN: uninformed; TF: treatment failure; TR: treatment response; ND: not determined; Score Joanna Briggs: classified as low risk of bias at 70% or more, medium risk of bias at 50-69%, and high risk of bias at 50% or less.^(17)^

**TABLE II:** Characteristics of selected studies that evaluated *Leishmania* genome mutations of genes associated with drug resistance (n = 9)

Author and year	*Leishmania* specie	Definition of resistance		Method	Evaluated genes	Drug-resistance associated mutation (N)
		Sensitive (N)	Resistant (N)			
Alizadeh R, 2011^(55)^	*L. tropica* *L. major*	UN (n = UN)	UN (n = UN)	Mutation screening/RFLP	*MDR* region	UN (97)
Adaui V, 2011b^(54)^	*L. braziliensis*	EC50 (n = 11)	EC50 (n = 10)	MLMT	Variations**MLMT	No association
Torres DC, 2013^(37)^	*L. braziliensis* *L. guyanensis*	Cure in three months (n = 29)	No cure in three months (n = 19)	Sanger sequencing	*AQP1*, *hsp70*, *MRPA* and *TRYR*	*hsp70*: *T579A*
Oliaee RT, 2018^(38)^	*L. tropica*	1 cycle with cure and no recurrence for six months (n = 14)	Active injuries after two cycles (n = 14)	Sanger sequencing	*AQP1*, γ-GCS, *MRPA*, *TDR1* and *TR*	No association
Rugani JN, 2019^64^	*L. braziliensis*	UN (n = 1)	UN (n = 2)	Sanger sequencing	*AQP1*	*AQP1*: *A516C*
Restrepo CM, 2019^(46)^	*L. panamensis*	EC50 (n = 2)	EC50 (n = 1)	Illumine sequencing (genoma)	Variations*	No association
Alijani Y, 2019^(19)^	*L. major*	Cure in three months (n = 145)	No cure in three months (n = 5)	PCR-RFLP	*AQP1*	*AQP1*: *G562A*
Fozongari F, 2020^(61)^	*L. tropica*	Cure with one cycle (n = 15)	No cure with one cycle (n = 15)	Sanger sequencing	Variations*	Four mutations could generate changes in the *TRYR* protein
Somee R, 2021^(20)^	*L. major*	Cure with one cycle (n = 5)	No cure with one cycle (n = 2)	Sanger sequencing	*AQP1*	*G700A*

PCR-RFLP: restriction fragment length polymorphism; MDR: multidrug drug region; MLMT: multilocus microsatellite typing; *AQP1*: Aquaglyceroporin 1; *MT*: Miltefosine transporter; γ-GCS: γ-glutamylcysteine synthetase; MRPA: multidrug resistance protein A; *TR*: Trypanothione reductase; *TDR1*: Thiol dependentreduxtase; *Hsp70*: shock proteins 70; UN: uninformed; NA: no association. *Estimation of chromosome some, detection of genetic amplifications, copy number variations, single nucleotide variations and indels over three par of bases; **Single nucleotide variations and phylogeny; EC50: median 50% effective concentration; N: number of sample.

**TABLE III:** Characteristics of selected studies that evaluated *Leishmania* expression of genes associated with drug resistance (n = 18)

Author and year	*Leishmania* species	Resistance rating		Rated gene	Resistance associated gene
		Sensitive (N)	Resistant (N)		
Torres, 2010^(31)^	*L. braziliensis* *L. guyanensis*	Cure in three months (n = 24)	No cure in three months (n = 13)	*AQP1, GSH1, TRYR, MRPA TDR1 E GSH2*	↑ γ-GCS *L. guyanensis* ↓*AQP1 L. braziliensis*
Adaui, 2011^(33)^	*L. braziliensis*	EC50 (n = 11)	EC50 (n = 10)	*ACR2, GSH2, MRPA, PABP, PAP14, S8, TDR1, META 1, TRYR, ODC, GSH1, AQP1*	↑ODC and TRYR
Kazemi-Rad, 2013b*^(56)^	*L. tropica*	Cure with three or less cycles (n = 1)	No cure with three or more cycles (n = 1)	Ubiquitina and *AAP3*	↑ Ubiquitina and AAP3
Kazemi-Rad, 2013a^(47)^	*L. tropica*	Cure with three or less cycles (n = 1)	No cure with three or more cycles (n = 1)	*AQP1, MAPK, (ABC) MRPA, PGK, PTP*	↑ MRPA, PTP and PGK. ↓ AQP1 e MAPK
Eslami, 2016^(62)^	*L. major*	Cure in three months (n = 12)	No cure in three months (n = 4)	*AQP1*	↓ AQP1
Ghobakhloo, 2016^(59)^	*L. major*	Cure in three months (n = 5)	No cure in three months (n = 5)	*AQP1, TDR-1, γ-GCS*	NA
Hajjaran, 2016^(58)^	*L.Tropica*	Cure with < 3 cycles (n = 18)	Three or more cycles without cure (n = 14)	*LACK1*	↓LACK1
Barrera, 2017^(13)^	*L. panamensis*	EC50 (n = 9)	EC50 (n = 10)	*abca2, abca3, abcc2, abcc3, abcg4, abcg6, AQP1, sams, sahh, B-tubulin*	↑ABCC3 ↓AQP1
Oliaee, 2018^(29)^	*L. tropica*	One cycle with cure and no recurrence for six months (n = 14)	Active injuries after two cycles (n = 14)	*AQP1, γ-GCS, MRPA, TDR1* and *TR*	↓ AQP1, γ-GCS and TDR1 ↑ MRPA, AQP1** γ-GCS**
Mohebali, 2019^(43)^	*L. Tropica*	Cure without recurrence in six months (n = 7)	Relapse in six months (n = 7)	γ-GCS, ODC, TRYR, AQP1 and *MRPA*	↑γ-GCS, TRYR and MRPA ↓AQP1
Ahmadian S, 2019^(60)^	*L. major*	Cure in three months (n = 9)	No cure in three months (n = 9)	*J-binding protein 1* and *2*	NA
Rugani, 2019^(18)^	*L. braziliensis*	UN (n = 1)	UN (n = 2)	*MRPA, AQP1, GSH1, ABCG2, ABCI4, AMR56, ARM58*	↓AQP1 ↑ARM58
Alijani Y, 2019^(19)^	*L. major*	Cure in three months (n = 145)	No cure in three months (n = 5)	*AQP1*	↑AQP1
Eslami G, 2021^(62)^	*L. major*	Cure with one cycle (n = 5)	No cure with one cycle (n = 5)	*AQP1*	↓AQP1
Somee R, 2021^(20)^	*L. major*	Cure with one cycle (n = 5)	No cure with one cycle (n = 2)	*AQP1* and *MAPK1*	↓AQP1 and ↓ MAPK1
Zabala-Pe**ñ**afiel, 2021^(36)^	*L. braziliensis*	Cure with one cycle (n = 5)	No cure with one cycle (n = 7)	serine proteases and tryparedoxin peroxidase transcripts46	NA
Nourbakhsh, 2021^(34)^	*L. major*	Cure with one cycle (n = 3)	No cure with one cycle (n = 5)	*LmTRYP, LmHSP 83, LmTRYR*	↓ LmTRYP, LmHSP 83, LmTRYR
Bahrami, 2022^(63)^	*L. tropica*	Cure with one cycle (n = 15)	Active injuries after three cycles (n = 10)	Iron-superoxide dismutases (*FeSOD*)	↑ FeSOD

*RNAseq; **Negative correlation between the level of expression of AQP1 and γ-GCS and the duration of the lesion in responsive patients (r = -1); MDR: multidrug drug region; *AQP1*: Aquaglyceroporin 1; *MT*: Miltefosine transporter; γ-GCS: γ-glutamylcysteine synthetase; *MRPA*: multidrug resistance protein A; *TR*: Trypanothione reductase; TDR1: Thiol dependent reduxtase; UN: uninformed; LACK1: *Leishmania*-activated C kinase gene; PGK: phosphoglycerate kinase; PTP: tyrosine phosphatase protein; AAP3: amino acid permease; ACR2: As/Sb Reductase; PABP: RNA-binding protein; ODC: ornithine decarboxylase; ROS3: complex Miltefosine transporter; META1: putative infective insect stage-specific Protein; S8: 40S ribosomal protein S8; PAP14: poly(A) polymerase; putative ABC: ABC-transporter; AMR56/58: antimony resistance marker; NA: No association; MAPK1: Mitogen-activated protein kinase.

Regarding the method used to obtain genetic material from *Leishmania* spp. all articles used biopsy or aspirated fluid. In six of these articles, drugs were also used in cultures to confirm parasite sensitivity and resistance. When the growth of the parasite in culture entered the logarithmic phase, the genetic material, DNA and/or RNA, was extracted. In one of the articles, the genetic material of the parasites was obtained directly from the aspirated fluid of the lesions. In eight (34.8%) articles, the genetic material was obtained directly from the biopsy sample, and in 15 other studies the parasites from biopsy cultures (Table I).

The species evaluated were *Leishmania (L.) tropica* in eight articles, *L. major* in eight, *L. braziliensis* in six, *L. panamensis* and *L. guyanensis* in two studies each. All cases investigated in the included articles were CL. Of all the articles, most investigated *Leishmania* gene expression (n = 18). Four of these articles investigated both mutation and gene expression. Five articles investigated only genomic mutations (Table I).

Of the nine studies that analysed mutations, only one examined the complete genome. The remaining eight studies analysed a total of eight different gene regions. Three mutations were identified in the Aquaglyceroporin 1 (*AQP1*) gene: A516C (*L. braziliensis*),^18^ G562A (*L. major*),^19^ and G700A (*L. major*).^20^ Additionally, a mutation in Heatshock 70 (*HSP70*) gene, T579A (*L. braziliensis*),^11^ was associated with drug resistance to pentavalent antimony. Although the T579A mutation in the *HSP70* gene was only identified in one article, this finding was strongly associated with a significant increase in the likelihood of treatment failure in *L. braziliensis* isolates. This mutation can predict 75% of cases of treatment failure with pentavalent antimonial, and this was the only included study in which the prediction of treatment outcome was calculated.^14^ One article identifies variations in the Trypanothione reductase (TRYR) gene that may affect the structure of the protein. This study used samples that were classified as resistant (no cure after one cycle of treatment), but it does not provide information on whether this mutation contributes to drug resistance. It suggests that further studies with different populations are needed to confirm resistance (Table II).

In the included studies, the gene expression associated with drug resistance was investigated in 30 different genes. Significantly increased expression was found in 13 genes: Multi-drug resistance protein A (*MRPA*) (n = 2 articles), ATP-binding cassette ABC transporters 3 (*ABCC3*) (n = 1 article), Gama synthetase (γ-GCS) (n = 2 articles), Antimony resistant marker (*ARM58*) (n = 1 article), *TRYR* (n = 2 articles), Ubiquitin (n = 1 article), *AAP3* (n = 1 article), *ODC* (ornithine decarboxylase) (n = 1 article), *AQP1* (n = 1 article), *FeSOD* (iron superoxide dismutases) (n = 1 article) and *AAP3* (n = 1 article). Eight genes were found to have a significant decrease in gene expression associated with resistance: *AQP1* (n = 8 articles), *MAPK* (Mitogen-Activated Protein Kinase) (n = 2 articles), *ROS3* (Complex Miltefosine Transporter) (n = 1 article), *GCS* (n = 1 article), *TRYR* (n = 1 article), *TRYP* (n = 1 article) and *HSP 83* (n = 1 article) (Table III). In most of the included articles, no measures of association such as relative risk (RR) and odds ratio (OR) for mutations in the genome and/or changes in parasite gene expression were calculated that would allow prediction of drug resistance.

## DISCUSSION

Drug resistance and treatment failure in leishmaniasis are challenging due to the limited number of available drugs and the lack of knowledge about therapeutic mechanisms of resistance, including genetic mechanisms.^4^ Resistance to *Leishmania* was studied primarily evaluated using antimonial pentavalent,^10^ one of the main drugs recommended by the WHO and widely used in Brazil^7^ and Iran.^21^ Leishmaniasis is a neglected disease that requires a comprehensive understanding of the molecular mechanisms of the parasite and the disease itself in order to effectively prevent and control it. With the development of advanced technologies, genome sequencing, gene expression profiling and the identification of genetic mutations and aneuploidies have become important public health tools to predict clinical outcomes, assessing drug-resistant strains and control the disease. This study is the first comprehensive effort to summarise the genes that may be associated with natural resistance to pentavalent antimony drug in *Leishmania*. Genes such as *AQP1*, *TDR1*, *TRYR*, *HSP70*, *FeSOD* and genes related to ABC transporters are potential markers for the prediction of drug resistance. By considering the genomic mutations and gene expression patterns of these genes, it is possible to improve the detection and prevention of drug resistance in *Leishmania*.

Of all the genes identified in this study, *AQP1* was the most extensively studied gene in relation to drug resistance in leishmaniasis. *AQP1* plays a crucial role in the osmotic regulation of *Leishmania* by facilitating the transport of water and unpaired polar solutes between the extracellular and intracellular environments.^22^ Additionally, *AQP1* is involved in the transport of various metabolites, including pentavalent antimonial, an important drug for the treatment of leishmaniasis.^23^ A decrease in *AQP1* gene expression, as observed in this review, would lead to reduced sensitivity to drugs.^4^ Furthermore, mutations in this gene could impair drug efflux and influx.^18^ Consequently, modulation of *AQP* genes can enhance susceptibility to the drug pentamidine, as has been demonstrated in resistant strains of *Trypanosoma bruce*i.^24^

The articles that assessed mutations in *AQP1* have demonstrated that a single change can modify the expression or function of the protein,^18^ ^,^ ^19^ ^,^ ^20^ leading to a disruption in solute transport.^25^ Several genetic variations have been identified, including one in *L. braziliensis* and two in *L. major*, all of which are associated with resistance to pentavalent antimony. These results suggest that strains of *Leishmania* spp. may undergo mutations and develop resistance to one of the most commonly used drugs for the treatment of cutaneous leishmaniasis, leading to a positive selection pressure for these strains. Additionally, a mutation in *AQP1* that induces similar resistance has also been described in *L. guyanensis* in an *in vitro* model.^26^ Therefore, epidemiologic and genomic surveillance is crucial for the identification of mutations and resistant strains.

Another significant protein described in the literature is TDR1, an enzyme present in parasites of the genus *Leishmania* and *Trypanosoma*. This protein is important for the control of redox regulation^27^ and is mainly expressed in the amastigote phase, which could explain the higher sensitivity of the mammalian phase to antimonials.^28^ The association between this gene and drug resistance may be species-specific, as the only study that reported an association investigated *L. tropica*.^29^ In *Trypanosoma cruzi*, application of the TDR1 protein has been shown to modulate the immune response against the parasite in mice, although similar results have not been found in *L. infantum*, a species associated with visceral forms.^30^ Therefore, further studies are needed to investigate the relationship between this gene and drug resistance.

The *TRYR* gene is another promising candidate for predicting treatment failure. This gene is conserved across species^11^ and is associated with thiol biosynthesis/redox metabolism.^31^ In trypanosomatids, trypanothione has been identified as a target for trivalent arsenic drugs.^32^ In this review, the results on *TRYR* expression were found to be inconsistent. Two articles found an increase in expression in *L. braziliensis* and *L. tropica*,^33^ and another found a decrease in expression associated with drug resistance in *L. major*.^34^ One difference between these studies is that different species were used, which emphasises the importance of further studies with different species to confirm the relevance of *TRYR* in treatment failure. When protein abundance and activity were assessed, *TRYR* activity was found to be overexpressed in antimony-resistant *L. tropica* strains.^35^ As this gene is parasite-specific, it is being studied more closely in drug development as it is unlikely to cause harm to the patient. In the search for new drugs against leishmaniasis, *TRYR* could serve as a therapeutic target or as a marker for resistant strains. Moreover, its use as a marker for drug resistance is facilitated by the fact that direct analysis of samples obtained from the lesion may be used, allowing faster and more appropriate intervention.

Other proteins that are part of the parasite’s defence against oxidative stress are also important, such as Glutathione synthetase, Spermidine Synthase, Trypanothione peroxidase, Trypanothione reductase Triparedoxin peroxidase. A higher expression of subtilisins and *TXNPx* genes was found in axenic amastigotes. This shows the phenotypic heterogeneity of the parasites.^36^ The control mechanisms of redox regulation are emerging as one of the main foci in the evaluation of drug resistance in parasites, as they represent a way for the parasite to avoid or reduce damage from the immune response, as shown in the articles included in this study.^35^ ^,^ ^36^ ^,^ ^37^ ^,^ ^38^ ^,^ ^39^

Among the genes identified in this review, *HSP70* can serve as a first line of defence against antimony.^40^ This may favour the phase shape change of *Leishmania* spp. and enable the infection of macrophages. In both *Schistosoma mansoni* ^41^ and *Plasmodium falciparum*,^42^ HSP70 is considered one of the most significant proteins involved in drug action. Although genetic errors that lead to mutations are a natural process, *HP70* is a highly conserved gene (0.00084 average pairwise nucleotide diversity) with a crucial function in the growth and life cycle of the parasite, and a mutation can impair its function. Therefore, mutations in this gene may disrupt these mechanisms and lead to increased resistance to the drug. Although the *T579A* mutation in the *HSP70* gene was identified in one article, this mutation may predict 75% of cases of treatment failure with pentavalent antimonial.^11^

In the present review, two articles indicated that an increase in *γ-GCS* gene expression was associated with resistance,^31^ ^,^ ^43^ while one article reported the opposite.^29^ Notably, these studies used the same methodology, investigated the same *Leishmania* species (*L. tropica*), were conducted in the same geographic region (Iran) and investigated the same drug for treatment.^29^ ^,^ ^43^ *γ-GCS* is an important enzyme involved in glutathione biosynthesis and plays a crucial role in cell defence against oxidative stress.^31^ ^,^ ^44^ Further studies are required to investigate the impact of the expression of this gene on the development of resistance. The *γ-GCS* is part of a group of proteins of the ABC transporter that appears to be important for the development of glucantim-resistant *Leishmania* strains. It was also found that in addition to the *GCS* gene, other genes such as *ODC* and *GSH1* were overexpressed in transfected *L. guyanensis* strains resistant to Glucantime.^45^ One article included in this review described increased expression of the *ODC* gene in association with drug resistance in *Leishmania* spp. Thus, an isolated analysis of the genes that constitute the ABC transporter does not suggest that they are suitable as reliable markers for drug resistance. However, an analysis considering all genes or a subset of them may contribute to a better prediction of drug resistance.

Another important gene is the gene encoding the MRPA protein, which is also part of the ABC transporter. In almost half of the studies in which MRPA expression was investigated, increased expression was associated with drug resistance. This can also impact the mechanism of pentavalent antimony influx or efflux, as antimony can be stored in intracellular vesicles of antimony-thiol complexes, leading to inactivation.^9^ ^,^ ^46^ Several studies have demonstrated that increased MRPA expression may be associated with drug resistance in *L. tropica*-induced injury.^18^ ^,^ ^29^ ^,^ ^43^ ^,^ ^47^ Similar observations have been made in other species such as *L. guyanensis*, *L. amazonensis* and *L. braziliensis* ^48^ as well as in other Kinetoplastids such as *T. brucei*.^49^ ^,^ ^50^

Iron transport is very important for *Leishmania*. FeSOD (iron superoxide dismutase) is a key to the antioxidant defence system of most organisms, including *Leishmania* parasites. SOD removes excess superoxide anions and converts them into hydrogen peroxide and oxygen.^51^ The response to oxidative stress caused by the immune system during defence is an important way to fight the parasite. The *Leishmania* use FeSOD to defend themselves against environmental detoxification and neutralise oxidative stress. In this review, Bahrami et al.^63^ shows an increased expression of FeSOD in most resistant samples. This adaptation within the cell is very important for resistant *Leishmania*.^52^

This systematic literature review focuses on the evaluation of clinically applicable data and excludes certain experimental aspects of drug resistance, such as studies using animal models, genetically modified strains or induction of gene overexpression. This exclusion may be considered a potential limitation of this study. However, the evaluation of genetic conditions of lesion samples would provide a better understanding of the real situation in cutaneous leishmaniasis lesions, including the real conditions for parasite adaptation to drugs and the mechanisms of immune response. Another limitation of the included studies is that most of them focused on specific genes, which limits their ability to provide a comprehensive understanding of the parasite’s transcriptome and complete genome changes. In addition, it should be noted that although the parasite was isolated from a culture of lesions in most of the included articles, this is not an ideal method for genetic evaluation compared to analysing material from biopsies, which would be more appropriate. In some articles, drug resistance was confirmed by *in vitro* drug susceptibility testing. However, *in vitro* drug susceptibility testing is not sufficient to detect treatment failure in leishmaniasis patients, as failure is multifactorial and *in vitro* results may not correlate with *in vivo* results. There is an urgent need to develop a more appropriate strategy to assess resistance.^53^ Nonetheless, articles using these analyses can help to identify genes associated with resistance that can be validated in future *in vivo* tests.^54^

Finally, there are no established guidelines for defining drug resistance in leishmaniasis treatment, and there is a lack of standardised methods for assessing resistance. This lack of standardisation hampers our understanding of susceptibility and resistance to antimonial pentavalent in leishmaniasis. Therefore, conducting studies with drug resistance classification guidelines and standardised assessment methods is important to obtain more reliable and applicable information.

Some mechanisms are crucial for the survival of *Leishmania* spp. Among the most important ones identified in this review are genes involved in defence against oxidative stress and conversion of the promastigote into an amastigote. Some of the genes associated with oxidative stress defence are *AQP1*, *TRYR*, *FeSOD* and *ABC* transporters, which are also important for parasite adaptation. *HSP70*, which is also discussed here, may play a significant role in the adaptation of the parasite to immune response.

The results of this study have important implications for the development of strategies against drug resistance in leishmaniasis. Understanding the genetic factors underlying drug resistance enables the design of targeted interventions and the development of new therapeutic approaches to address this challenge. It also facilitates the development of markers to assess drug resistance, enabling more effective treatment. Continued research in this area and further development of molecular technologies will further improve our ability to combat drug resistance and effectively treat leishmaniasis.
