# Supplementary material for: Leishmania spp. genetic factors associated with cutaneous leishmaniasis antimony pentavalent drug resistance: a systematic review
Source: Mem Inst Oswaldo Cruz. 2024 Sep 2;119:e230240. doi: 10.1590/0074-02760230240 (PMC11370656; doi:10.1590/0074-02760230240)
Supplement: Supplementary file 1 [file 1678-8060-mioc-119-e230240-s.pdf]

TABLE I

PRISMA checklist of the systematic review guidelines

Section and topic	Item	Checklist item	Location where item is reported
TITLE			
Title	1	Identify the report as a systematic review.	p1; 1 - 2
ABSTRACT			
Abstract	2	See the PRISMA 2020 for Abstracts checklist.	Supplementary Table II
INTRODUCTION			
Rationale	3	Describe the rationale for the review in the context of existing knowledge.	p6; 128 - 141
Objectives	4	Provide an explicit statement of the objective(s) or question(s) the review addresses.	p6; 141 - 143
METHODS			
Eligibility criteria	5	Specify the inclusion and exclusion criteria for the review and how studies were grouped for the syntheses.	p7; 162 - 187
Information sources	6	Specify all databases, registers, websites, organisations, reference lists and other sources searched or consulted to identify studies. Specify the date when each source was last searched or consulted.	p7; 152 - 154
Search strategy	7	Present the full search strategies for all databases, registers and websites, including any filters and limits used.	p7; 152 - 160
Selection process	8	Specify the methods used to decide whether a study met the inclusion criteria of the review, including how many reviewers screened each record and each report retrieved, whether they worked independently, and if applicable, details of automation tools used in the process.	p8; 177 - 180
Data collection process	9	Specify the methods used to collect data from reports, including how many reviewers collected data from each report, whether they worked independently, any processes for obtaining or confirming data from study investigators, and if applicable, details of automation tools used in the process.	p8; 182 - 187
Data items	10a	List and define all outcomes for which data were sought. Specify whether all results that were compatible with each outcome domain in each study were sought (e.g. for all measures, time points, analyses), and if not, the methods used to decide which results to collect.	p8; 182 - 187
	10b	List and define all other variables for which data were sought (e.g. participant and intervention characteristics, funding sources). Describe any assumptions made about any missing or unclear information.	p8; 182 - 187
Study risk of bias assessment	11	Specify the methods used to assess risk of bias in the included studies, including details of the tool(s) used, how many reviewers assessed each study and whether they worked independently, and if applicable, details of automation tools used in the process.	p8; 189 - 193
Effect measures	12	Specify for each outcome the effect measure(s) (e.g. risk ratio, mean difference) used in the synthesis or presentation of results.	p9; 210 - 222
Synthesis methods	13a	Describe the processes used to decide which studies were eligible for each synthesis (e.g. tabulating the study intervention characteristics and comparing against the planned groups for each synthesis (item #5)).	p7; 162 - 174
	13b	Describe any methods required to prepare the data for presentation or synthesis, such as handling of missing summary statistics, or data conversions.	p8; 182 - 187
	13c	Describe any methods used to tabulate or visually display results of individual studies and syntheses.	p8; 182 - 187
	13d	Describe any methods used to synthesise results and provide a rationale for the choice(s). If meta-analysis was performed, describe the model(s), method(s) to identify the presence and extent of statistical heterogeneity, and software package(s) used.	p8; 182 - 187
	13e	Describe any methods used to explore possible causes of heterogeneity among study results (e.g. subgroup analysis, meta-regression).	NA
	13f	Describe any sensitivity analyses conducted to assess robustness of the synthesised results.	NA
Reporting bias assessment	14	Describe any methods used to assess risk of bias due to missing results in a synthesis (arising from reporting biases).	p8; 189 - 193
Certainty assessment	15	Describe any methods used to assess certainty (or confidence) in the body of evidence for an outcome.	p8; 189 - 193 and p11; 249-251
RESULTS			
Study selection	16a	Describe the results of the search and selection process, from the number of records identified in the search to the number of studies included in the review, ideally using a flow diagram.	p9; 204 - 208
	16b	Cite studies that might appear to meet the inclusion criteria, but which were excluded, and explain why they were excluded.	p7; 169 - 174
Study characteristics	17	Cite each included study and present its characteristics.	p9; 210 – 222<br>p10; 224 – 230<br>p10; 232 – 236<br>p10; 238 – 251<br>p11; 253 - 265

CC BY

Section and topic	Item	Checklist item	Location where item is reported
Risk of bias in studies	18	Present assessments of risk of bias for each included study.	p9; 207 - 208
Results of individual studies	19	For all outcomes, present, for each study: (a) summary statistics for each group (where appropriate) and (b) an effect estimate and its precision (e.g. confidence/credible interval), ideally using structured tables or plots.	p10; 238 - 265
Results of syntheses	20a	For each synthesis, briefly summarise the characteristics and risk of bias among contributing studies.	p24 and 25
	20b	Present results of all statistical syntheses conducted. If meta-analysis was done, present for each the summary estimate and its precision (e.g. confidence/credible interval) and measures of statistical heterogeneity. If comparing groups, describe the direction of the effect.	p10; 238 - 265
	20c	Present results of all investigations of possible causes of heterogeneity among study results.	NA
	20d	Present results of all sensitivity analyses conducted to assess the robustness of the synthesised results.	NA
Reporting biases	21	Present assessments of risk of bias due to missing results (arising from reporting biases) for each synthesis assessed.	p24 and 25
Certainty of evidence	22	Present assessments of certainty (or confidence) in the body of evidence for each outcome assessed.	p10; 238 - 265
DISCUSSION			
Discussion	23a	Provide a general interpretation of the results in the context of other evidence.	p12; 269 - 387
	23b	Discuss any limitations of the evidence included in the review.	p17; 389 - 406
	23c	Discuss any limitations of the review processes used.	p17; 389 - 406
	23d	Discuss implications of the results for practice, policy, and future research.	p18; 422 - 428
OTHER INFORMATION			
Registration and protocol	24a	Provide registration information for the review, including register name and registration number, or state that the review was not registered.	p9; 195 - 200
	24b	Indicate where the review protocol can be accessed, or state that a protocol was not prepared.	p9; 195 - 200
	24c	Describe and explain any amendments to information provided at registration or in the protocol.	p9; 195 - 200
Support	25	Describe sources of financial or non-financial support for the review, and the role of the funders or sponsors in the review.	p18; 430 - 434
Competing interests	26	Declare any competing interests of review authors.	p19; 437
Availability of data, code and other materials	27	Report which of the following are publicly available and where they can be found: template data collection forms; data extracted from included studies; data used for all analyses; analytic code; any other materials used in the review.	p9; 195 - 200

From: Page MJ, McKenzie JE, Bossuyt PM, Boutron I, Hoffmann TC, Mulrow CD, et al. The PRISMA 2020 statement: an updated guideline for reporting systematic reviews. BMJ 2021; 372: n71. doi: 10.1136/bmj.n71. NA: not applicable (section for met-analyses only).

TABLE II

PRISMA abstract checklist of the systematic review guidelines

Section and topic	Item	Checklist item	Reported (Yes/No)
TITLE			
Title	1	Identify the report as a systematic review.	p1, 1-2
BACKGROUND			
Objectives	2	Provide an explicit statement of the main objective(s) or question(s) the review addresses.	p2, 35-37
METHODS			
Eligibility criteria	3	Specify the inclusion and exclusion criteria for the review.	p2, 38-41
Information sources	4	Specify the information sources (e.g. databases, registers) used to identify studies and the date when each was last searched.	p2, 37-38
Risk of bias	5	Specify the methods used to assess risk of bias in the included studies.	p2, 41-42
Synthesis of results	6	Specify the methods used to present and synthesise results.	p2, 37-38
RESULTS			
Included studies	7	Give the total number of included studies and participants and summarise relevant characteristics of studies.	p2, 42-51
Synthesis of results	8	Present results for main outcomes, preferably indicating the number of included studies and participants for each. If meta-analysis was done, report the summary estimate and confidence/credible interval. If comparing groups, indicate the direction of the effect (i.e. which group is favoured).	p2, 42-48
DISCUSSION			
Limitations of evidence	9	Provide a brief summary of the limitations of the evidence included in the review (e.g. study risk of bias, inconsistency and imprecision).	p3, 48-51
Interpretation	10	Provide a general interpretation of the results and important implications.	p3, 51- 53
OTHER			
Funding	11	Specify the primary source of funding for the review.	p3, 58-61
Registration	12	Provide the register name and registration number.	p3, 63-64

From: Page MJ, McKenzie JE, Bossuyt PM, Boutron I, Hoffmann TC, Mulrow CD, et al. The PRISMA 2020 statement: an updated guideline for reporting systematic reviews. BMJ 2021; 372: n71. doi: 10.1136/bmj.n71

Joanna Briggs Institute Critical Appraisal Checklist for each study included in this systematic review

JBI Critical Appraisal Checklist for Analytical Cross Sectional Studies
Reviewer: RLAN
Date: March, 13, 2023
Authors: Torres DC, Adaui V, Ribeiro-Alves M, Romero GA, Arévalo J, Cupolillo E, Dujardin JC. Year: 2010
Record number: https://pubmed.ncbi.nlm.nih.gov/20478409/

	Yes	No	Unclear	Not applicable
1. Were the criteria for inclusion in the sample clearly defined?	X	□	□	□
2. Were the study subjects and the setting described in detail?	X	□	□	□
3. Was the exposure measured in a valid and reliable way?	X	□	□	□
4. Were objective, standard criteria used for measurement of the condition?	X	□	□	□
5. Were confounding factors identified?	X	□	□	□
6. Were strategies to deal with confounding factors stated?	X	□	□	□
7. Were the outcomes measured in a valid and reliable way?	X	□	□	□
8. Was appropriate statistical analysis used?	X	□	□	□

Overall appraisal: Include **(X)** Exclude ( ) Seek further info ( )
Score: 100%
Comments: Not applicable.

JBI Critical Appraisal Checklist for Analytical Cross Sectional Studies
Reviewer: RLAN
Date: March, 13, 2023
Authors: Adaui V, Schnorbusch K, Zimic M, Gutiérrez A, Decuypere S, Vanaerschot M, DE Doncker S, Maes I, Llanos-Cuentas A, Chappuis F, Arévalo J, Dujardin JC.
Year: 2011
Record number: https://pubmed.ncbi.nlm.nih.gov/20678296/

	Yes	No	Unclear	Not applicable
1. Were the criteria for inclusion in the sample clearly defined?	X	□	□	□
2. Were the study subjects and the setting described in detail?	X	□	□	□
3. Was the exposure measured in a valid and reliable way?	X	□	□	□
4. Were objective, standard criteria used for measurement of the condition?	X	□	□	□
5. Were confounding factors identified?	X	□	□	□
6. Were strategies to deal with confounding factors stated?	X	□	□	□
7. Were the outcomes measured in a valid and reliable way?	X	□	□	□
8. Was appropriate statistical analysis used?	X	□	□	□

Overall appraisal: Include **(X)** Exclude ( ) Seek further info ( )
Score: 100%
Comments: Not applicable.

JBI Critical Appraisal Checklist for Cohort Studies
Reviewer: RLAN
Date: March, 13, 2023
Authors: Adaui V, Maes I, Huyse T, Van den Broeck F, Talledo M, Kuhls K, De Doncker S, Maes L, Llanos-Cuentas A, Schönian G, Arevalo J, Dujardin JC.
Year: 2011
Record Number: https://pubmed.ncbi.nlm.nih.gov/21871584/

	Yes	No	Unclear	Not applicable
1. Were the two groups similar and recruited from the same population?	X	□	□	□
2. Were the exposures measured similarly to assign people to both exposed and unexposed groups?	X	□	□	□
3. Was the exposure measured in a valid and reliable way?	X	□	□	□
4. Were confounding factors identified?	X	□	□	□
5. Were strategies to deal with confounding factors stated?	X	□	□	□
6. Were the groups/participants free of the outcome at the start of the study (or at the moment of exposure)?	X	□	□	□
7. Were the outcomes measured in a valid and reliable way?	X	□	□	□
8. Was the follow up time reported and sufficient to be long enough for outcomes to occur?	X	□	□	□
9. Was follow up complete, and if not, were the reasons to loss to follow up described and explored?	X	□	□	□
10. Were strategies to address incomplete follow up utilised?	□	□	X	□
11. Was appropriate statistical analysis used?	X	□	□	□

Overall appraisal: Include **(X)** Exclude ( ) Seek further info ( )
Score: 90.9%
Comments: Overall appraisal

JBI Critical Appraisal Checklist for Analytical Cross Sectional Studies
Reviewer: RLAN
Date: March, 13, 2023
Author: Alizadeh R, Hooshyar H, Bandehpor M, Arbabi M, Kazemi F, Talari A, Kazemi B.
Year: 2011
Record number: https://pubmed.ncbi.nlm.nih.gov/22737430/

	Yes	No	Unclear	Not applicable
1. Were the criteria for inclusion in the sample clearly defined?	X	□	□	□
2. Were the study subjects and the setting described in detail?	X	□	□	□
3. Was the exposure measured in a valid and reliable way?	X	□	□	□
4. Were objective, standard criteria used for measurement of the condition?	X	□	□	□
5. Were confounding factors identified?	X	□	□	□
6. Were strategies to deal with confounding factors stated?	X	□	□	□
7. Were the outcomes measured in a valid and reliable way?	X	□	□	□
8. Was appropriate statistical analysis used?	X	□	□	□

Overall appraisal: Include **(X)** Exclude ( ) Seek further info ( )
Score: 100%
Comments: Not applicable

JBI Critical Appraisal Checklist for Analytical Cross Sectional Studies
Reviewer: RLAN, TMSS
Date: August, 09, 2023
Authors: Kazemi-Rad E, Mohebali M, Khadem-Erfan MB, Saffari M, Raoofian R, Hajjaran H, Hadighi R, Khamesipour A, Rezaie S, Abedkhojasteh H, Heidari M.
Year: 2013
Record number: https://pubmed.ncbi.nlm.nih.gov/23928349/

	Yes	No	Unclear	Not applicable
1. Were the criteria for inclusion in the sample clearly defined?	X	□	□	□
2. Were the study subjects and the setting described in detail?	X	□	□	□
3. Was the exposure measured in a valid and reliable way?	X	□	□	□
4. Were objective, standard criteria used for measurement of the condition?	X	□	□	□
5. Were confounding factors identified?	X	□	□	□
6. Were strategies to deal with confounding factors stated?	X	□	□	□
7. Were the outcomes measured in a valid and reliable way?	X	□	□	□
8. Was appropriate statistical analysis used?	X	□	□	□

Overall appraisal: Include **(X)** Exclude ( ) Seek further info ( )
Score: 100%
Comments: Not applicable

JBI Critical Appraisal Checklist for Analytical Cross Sectional Studies
Reviewer: RLAN, TMSS
Date: August, 09, 2023
Authors: Kazemi-Rad E, Mohebali M, Khadem-Erfan MB, Hajjaran H, Hadighi R, Khamesipour A, Rezaie S, Saffari M, Raoofian R, Heidari M.
Year: 2013
Record number: https://pubmed.ncbi.nlm.nih.gov/24039283/

	Yes	No	Unclear	Not applicable
1. Were the criteria for inclusion in the sample clearly defined?	X	□	□	□
2. Were the study subjects and the setting described in detail?	X	□	□	□
3. Was the exposure measured in a valid and reliable way?	X	□	□	□
4. Were objective, standard criteria used for measurement of the condition?	X	□	□	□
5. Were confounding factors identified?	X	□	□	□
6. Were strategies to deal with confounding factors stated?	X	□	□	□
7. Were the outcomes measured in a valid and reliable way?	X	□	□	□
8. Was appropriate statistical analysis used?	X	□	□	□

Overall appraisal: Include **(X)** Exclude ( ) Seek further info ( )
Score: 100%
Comments: Not applicable

JBI Critical Appraisal Checklist for Cohort Studies
Reviewer: RLAN, TMSS
Date: March, 13, 2023
Authors: Torres DC, Ribeiro-Alves M, Romero GA, Dávila AM, Cupolillo E.
Year: 2013
Record number: https://www.sciencedirect.com/science/article/pii/S0001706X13000260

	Yes	No	Unclear	Not applicable
1. Were the two groups similar and recruited from the same population?	X	□	□	□
2. Were the exposures measured similarly to assign people to both exposed and unexposed groups?	X	□	□	□
3. Was the exposure measured in a valid and reliable way?	X	□	□	□
4. Were confounding factors identified?	X	□	□	□
5. Were strategies to deal with confounding factors stated?	X	□	□	□
6. Were the groups/participants free of the outcome at the start of the study (or at the moment of exposure)?	X	□	□	□
7. Were the outcomes measured in a valid and reliable way?	X	□	□	□
8. Was the follow up time reported and sufficient to be long enough for outcomes to occur?	X	□	□	□
9. Was follow up complete, and if not, were the reasons to loss to follow up described and explored?	X	□	□	□
10. Were strategies to address incomplete follow up utilised?	□	X	□	□
11. Was appropriate statistical analysis used?	X	□	□	□

Overall appraisal: Include **(X)** Exclude ( ) Seek further info ( )
Score: 90.9%
Comments: Overall appraisal

JBI Critical Appraisal Checklist for Cohort Studies
Reviewer: RLAN, TMSS
Date: March, 13, 2023
Authors: Eslami G, Zarchi MV, Moradi A, Hejazi SH, Sohrevardi SM, Vakili M, Khamesipour A.
Year: 2016
Record number: https://pubmed.ncbi.nlm.nih.gov/28035115/

	Yes	No	Unclear	Not applicable
1. Were the two groups similar and recruited from the same population?	X	□	□	□
2. Were the exposures measured similarly to assign people to both exposed and unexposed groups?	X	□	□	□
3. Was the exposure measured in a valid and reliable way?	X	□	□	□
4. Were confounding factors identified?	X	□	□	□
5. Were strategies to deal with confounding factors stated?	X	□	□	□
6. Were the groups/participants free of the outcome at the start of the study (or at the moment of exposure)?	X	□	□	□
7. Were the outcomes measured in a valid and reliable way?	X	□	□	□
8. Was the follow up time reported and sufficient to be long enough for outcomes to occur?	X	□	□	□
9. Was follow up complete, and if not, were the reasons to loss to follow up described and explored?	X	□	□	□
10. Were strategies to address incomplete follow up utilised?	X	□	□	□
11. Was appropriate statistical analysis used?	X	□	□	□

Overall appraisal: Include **(X)** Exclude ( ) Seek further info ( )
Score: 100%
Comments: Overall appraisal

JBI Critical Appraisal Checklist for Analytical Cross Sectional Studies
Reviewer: RLAN
Date: March, 13, 2023
Authors: Hajjaran H, Kazemi-Rad E, Mohebali M, Oshaghi MA, Khadem-Erfan MB, Hajaliloo E, Reisi Nafchi H, Raoofian R.
Year: 2016
Record number: https://pubmed.ncbi.nlm.nih.gov/27336481/

	Yes	No	Unclear	Not applicable
1. Were the criteria for inclusion in the sample clearly defined?	X	□	□	□
2. Were the study subjects and the setting described in detail?	X	□	□	□
3. Was the exposure measured in a valid and reliable way?	X	□	□	□
4. Were objective, standard criteria used for measurement of the condition?	X	□	□	□
5. Were confounding factors identified?	X	□	□	□
6. Were strategies to deal with confounding factors stated?	X	□	□	□
7. Were the outcomes measured in a valid and reliable way?	X	□	□	□
8. Was appropriate statistical analysis used?	X	□	□	□

Overall appraisal: Include **(X)** Exclude ( ) Seek further info ( )
Score: 100%
Comments: Not applicable

JBI Critical Appraisal Checklist for Cohort Studies
Reviewer: RLAN
Date: March, 13, 2023
Authors: Ghobakhloo N, Motazedian MH, Fardaei M..
Year: 2016
Record number: https://pubmed.ncbi.nlm.nih.gov/31803775/

	Yes	No	Unclear	Not applicable
1. Were the two groups similar and recruited from the same population?	X	□	□	□
2. Were the exposures measured similarly to assign people to both exposed and unexposed groups?	X	□	□	□
3. Was the exposure measured in a valid and reliable way?	X	□	□	□
4. Were confounding factors identified?	X	□	□	□
5. Were strategies to deal with confounding factors stated?	X	□	□	□
6. Were the groups/participants free of the outcome at the start of the study (or at the moment of exposure)?	X	□	□	□
7. Were the outcomes measured in a valid and reliable way?	X	□	□	□
8. Was the follow up time reported and sufficient to be long enough for outcomes to occur?	X	□	□	□
9. Was follow up complete, and if not, were the reasons to loss to follow up described and explored?	X	□	□	□
10. Were strategies to address incomplete follow up utilised?	□	X	□	□
11. Was appropriate statistical analysis used?	X	□	□	□

Overall appraisal: Include **(X)** Exclude ( ) Seek further info ( )
Score: 90.9%
Comments: Overall appraisal

JBI Critical Appraisal Checklist for Analytical Cross Sectional Studies
Reviewer: RLAN
Date: March, 13, 2023
Authors: Barrera MC, Rojas LJ, Weiss A, Fernandez O, McMahon-Pratt D, Saravia NG, Gomez MA.
Year: 2017
Record number: https://pubmed.ncbi.nlm.nih.gov/28843396/

	Yes	No	Unclear	Not applicable
1. Were the criteria for inclusion in the sample clearly defined?	X	□	□	□
2. Were the study subjects and the setting described in detail?	X	□	□	□
3. Was the exposure measured in a valid and reliable way?	X	□	□	□
4. Were objective, standard criteria used for measurement of the condition?	X	□	□	□
5. Were confounding factors identified?	□	X	□	□
6. Were strategies to deal with confounding factors stated?	X	□	□	□
7. Were the outcomes measured in a valid and reliable way?	X	□	□	□
8. Was appropriate statistical analysis used?	X	□	□	□

Overall appraisal: Include **(X)** Exclude ( ) Seek further info ( )
Score: 87.5%
Comments: Not applicable

JBI Critical Appraisal Checklist for Cohort Studies
Reviewer: RLAN
Date: March, 13, 2023
Authors: Oliaee RT, Sharifi I, Afgar A, Kareshk AT, Asadi A, Heshmatkhah A, Bamorovat M, Jafarzadeh A, Mohammadi MA, Daneshvar H.
Year: 2018
Record number: https://doi.org/10.1111/tmi.13062

	Yes	No	Unclear	Not applicable
1. Were the two groups similar and recruited from the same population?	X	□	□	□
2. Were the exposures measured similarly to assign people to both exposed and unexposed groups?	X	□	□	□
3. Was the exposure measured in a valid and reliable way?	X	□	□	□
4. Were confounding factors identified?	X	□	□	□
5. Were strategies to deal with confounding factors stated?	X	□	□	□
6. Were the groups/participants free of the outcome at the start of the study (or at the moment of exposure)?	X	□	□	□
7. Were the outcomes measured in a valid and reliable way?	X	□	□	□
8. Was the follow up time reported and sufficient to be long enough for outcomes to occur?	X	□	□	□
9. Was follow up complete, and if not, were the reasons to loss to follow up described and explored?	X	□	□	□
10. Were strategies to address incomplete follow up utilised?	□	□	X	□
11. Was appropriate statistical analysis used?	X	□	□	□

Overall appraisal: Include **(X)** Exclude ( ) Seek further info ( )
Score: 90.9%
Comments: Overall appraisal

JBI Critical Appraisal Checklist for Analytical Cross Sectional Studies
Reviewer: RLAN
Date: March, 13, 2023
Authors: Rugani JN, Gontijo CMF, Frézard F, Soares RP, Monte-Neto RLD.
Year: 2019
Record number: https://pubmed.ncbi.nlm.nih.gov/31433006/

	Yes	No	Unclear	Not applicable
1. Were the criteria for inclusion in the sample clearly defined?	X	□	□	□
2. Were the study subjects and the setting described in detail?	X	□	□	□
3. Was the exposure measured in a valid and reliable way?	X	□	□	□
4. Were objective, standard criteria used for measurement of the condition?	X	□	□	□
5. Were confounding factors identified?	X	□	□	□
6. Were strategies to deal with confounding factors stated?	X	□	□	□
7. Were the outcomes measured in a valid and reliable way?	X	□	□	□
8. Was appropriate statistical analysis used?	X	□	□	□

Overall appraisal: Include **(X)** Exclude ( ) Seek further info ( )
Score: 100%
Comments: Not applicable

JBI Critical Appraisal Checklist for Analytical Cross Sectional Studies
Reviewer: RLAN
Date: March, 13, 2023
Authors: Mohebali M, Kazemirad E, Hajjaran H, Kazemirad E, Oshaghi MA, Raoofian R, Teimouri A.
Year: 2019
Record number: https://pubmed.ncbi.nlm.nih.gov/30390113/

	Yes	No	Unclear	Not applicable
1. Were the criteria for inclusion in the sample clearly defined?	X	□	□	□
2. Were the study subjects and the setting described in detail?	X			□
3. Was the exposure measured in a valid and reliable way?	X	□		□
4. Were objective, standard criteria used for measurement of the condition?	X	□		□
5. Were confounding factors identified?	X	□		□
6. Were strategies to deal with confounding factors stated?	X	□		□
7. Were the outcomes measured in a valid and reliable way?	X	□		□
8. Was appropriate statistical analysis used?	X	□		□

Overall appraisal: Include **(X)** Exclude ( ) Seek further info ( )
Score: 100%
Comments: Not applicable

JBI Critical Appraisal Checklist for Analytical Cross Sectional Studies
Reviewer: RLAN
Date: March, 13, 2023
Authors: Alijani Y, Hosseini SS, Ahmadian S, Boughattas S, Eslami G, Naderian S, Ajamein V.
Year: 2019
Record number: https://pubmed.ncbi.nlm.nih.gov/31803775/

	Yes	No	Unclear	Not applicable
1. Were the criteria for inclusion in the sample clearly defined?	X	□	□	□
2. Were the study subjects and the setting described in detail?	X	□	□	□
3. Was the exposure measured in a valid and reliable way?	X	□	□	□
4. Were objective, standard criteria used for measurement of the condition?	X	□	□	□
5. Were confounding factors identified?	X	□	□	□
6. Were strategies to deal with confounding factors stated?	X	□	□	□
7. Were the outcomes measured in a valid and reliable way?	X	□	□	□
8. Was appropriate statistical analysis used?	X	□	□	□

Overall appraisal: Include **(X)** Exclude ( ) Seek further info ( )
Score: 100%
Comments: Not applicable

JBI Critical Appraisal Checklist for Cohort Studies
Reviewer: RLAN
Date: March, 13, 2023
Authors: Ahmadian S, Eslami G, Fatahi A, Hosseini SS, Vakili M, Ajamein Fahadan V, Elloumi M.
Year: 2019
Record number: https://pubmed.ncbi.nlm.nih.gov/29709098/

	Yes	No	Unclear	Not applicable
1. Were the two groups similar and recruited from the same population?	X	□	□	□
2. Were the exposures measured similarly to assign people to both exposed and unexposed groups?	X	□	□	□
3. Was the exposure measured in a valid and reliable way?	X	□	□	□
4. Were confounding factors identified?	X	□	□	□
5. Were strategies to deal with confounding factors stated?	X	□	□	□
6. Were the groups/participants free of the outcome at the start of the study (or at the moment of exposure)?	X	□	□	□
7. Were the outcomes measured in a valid and reliable way?	X	□	□	□
8. Was the follow up time reported and sufficient to be long enough for outcomes to occur?	X	□	□	□
9. Was follow up complete, and if not, were the reasons to loss to follow up described and explored?	X	□	□	□
10. Were strategies to address incomplete follow up utilised?	□	□	X	□
11. Was appropriate statistical analysis used?	X	□	□	□

Overall appraisal: Include **(X)** Exclude ( ) Seek further info ( )
Score: 90.9%
Comments: Overall appraisal

JBI Critical Appraisal Checklist for Analytical Cross Sectional Studies
Reviewer: RLAN
Date: March, 13, 2023
Authors: Restrepo CM, Llanes A, Cedeño EM, Chang JH, Álvarez J, Ríos M, Penagos H, Suárez JA, Lleonart R.
Year: 2019
Record number: https://pubmed.ncbi.nlm.nih.gov/31652919/

	Yes	No	Unclear	Not applicable
1. Were the criteria for inclusion in the sample clearly defined?	X	□	□	□
2. Were the study subjects and the setting described in detail?	X	□	□	□
3. Was the exposure measured in a valid and reliable way?	X	□	□	□
4. Were objective, standard criteria used for measurement of the condition?	X	□	□	□
5. Were confounding factors identified?	X	□	□	□
6. Were strategies to deal with confounding factors stated?	X	□	□	□
7. Were the outcomes measured in a valid and reliable way?	X	□	□	□
8. Was appropriate statistical analysis used?	X	□	□	□

Overall appraisal: Include **(X)** Exclude ( ) Seek further info ( )
Score: 100%
Comments: Not applicable

JBI Critical Appraisal Checklist for Analytical Cross Sectional Studies
Reviewer: RLAN
Date: March, 13, 2023
Authors: Fozongari F, Dalimi A, Arab SS, Behmanesh M, Khammari A.
Year: 2020
Record number: https://pubmed.ncbi.nlm.nih.gov/33884008/

	Yes	No	Unclear	Not applicable
1. Were the criteria for inclusion in the sample clearly defined?	X	□	□	□
2. Were the study subjects and the setting described in detail?	X	□	□	□
3. Was the exposure measured in a valid and reliable way?	X	□	□	□
4. Were objective, standard criteria used for measurement of the condition?	X	□	□	□
5. Were confounding factors identified?	X	□	□	□
6. Were strategies to deal with confounding factors stated?	X	□	□	□
7. Were the outcomes measured in a valid and reliable way?	X	□	□	□
8. Was appropriate statistical analysis used?	X	□	□	□

Overall appraisal: Include **(X)** Exclude ( ) Seek further info ( )
Score: 100%
Comments: Not applicable

JBI Critical Appraisal Checklist for Analytical Cross Sectional Studies
Reviewer: RLAN
Date: March, 13, 2023
Authors: Eslami G, Hatefi S, Ramezani V, Tohidfar M, Churkina TV, Orlov YL, Hosseini SS, Boozhmehrani MJ, Vakili M.
Year: 2021
Record number: https://pubmed.ncbi.nlm.nih.gov/33763300/

	Yes	No	Unclear	Not applicable
1. Were the criteria for inclusion in the sample clearly defined?	X	□	□	□
2. Were the study subjects and the setting described in detail?	X	□	□	□
3. Was the exposure measured in a valid and reliable way?	X	□	□	□
4. Were objective, standard criteria used for measurement of the condition?	X	□	□	□
5. Were confounding factors identified?	X	□	□	□
6. Were strategies to deal with confounding factors stated?	X	□	□	□
7. Were the outcomes measured in a valid and reliable way?	X	□	□	□
8. Was appropriate statistical analysis used?	X	□	□	□

Overall appraisal: Include **(X)** Exclude ( ) Seek further info ( )
Score: 100%
Comments: Not applicable

JBI Critical Appraisal Checklist for Analytical Cross Sectional Studies
Reviewer: RLAN
Date: March, 13, 2023
Authors: Somee R, Eslami G, Vakili M.
Year: 2021
Record number: https://pubmed.ncbi.nlm.nih.gov/34415479/

	Yes	No	Unclear	Not applicable
1. Were the criteria for inclusion in the sample clearly defined?	X	□	□	□
2. Were the study subjects and the setting described in detail?	X	□	□	□
3. Was the exposure measured in a valid and reliable way?	X	□	□	□
4. Were objective, standard criteria used for measurement of the condition?	X	□	□	□
5. Were confounding factors identified?	X	□	□	□
6. Were strategies to deal with confounding factors stated?	X	□	□	□
7. Were the outcomes measured in a valid and reliable way?	X	□	□	□
8. Was appropriate statistical analysis used?	X	□	□	□

Overall appraisal: Include **(X)** Exclude ( ) Seek further info ( )
Score: 100%
Comments: Not applicable

JBI Critical Appraisal Checklist for Analytical Cross Sectional Studies
Reviewer: RLAN
Date: March, 13, 2023
Authors: Anabel Zabala-Peñafiel1, Geovane Dias-Lopes1, Léa Cysne-Finkelstein2, Fátima Conceição-Silva2, Luciana de Freitas Campos Miranda3, Aline Fagundes3, Armando de Oliveira Schubach3, Maria Inês Fernandes Pimentel3, Franklin Souza-Silva1, Lucas de Almeida Machado4 & Carlos Roberto Alves1
Year: 2021
Record number: https://www.nature.com/articles/s41598-021-93665-z

	Yes	No	Unclear	Not applicable
1. Were the criteria for inclusion in the sample clearly defined?	X	□	□	□
2. Were the study subjects and the setting described in detail?	X	□	□	□
3. Was the exposure measured in a valid and reliable way?	X	□	□	□
4. Were objective, standard criteria used for measurement of the condition?	X	□	□	□
5. Were confounding factors identified?	X	□	□	□
6. Were strategies to deal with confounding factors stated?	X	□	□	□
7. Were the outcomes measured in a valid and reliable way?	X	□	□	□
8. Was appropriate statistical analysis used?	X	□	□	□

Overall appraisal: Include **(X)** Exclude ( ) Seek further info ( )
Score: 100%
Comments: Not applicable

JBI Critical Appraisal Checklist for Analytical Cross Sectional Studies
Reviewer: RLAN
Date: March, 13, 2023
Authors: Aida NOURBAKHSH, Gilda ESLAMI, Seyed Mojtaba SOHREVARDI, Mahmood VAKILI
Year: 2021
Record number: https://pubmed.ncbi.nlm.nih.gov/35294143/

	Yes	No	Unclear	Not applicable
1. Were the criteria for inclusion in the sample clearly defined?	X	□	□	□
2. Were the study subjects and the setting described in detail?	X	□	□	□
3. Was the exposure measured in a valid and reliable way?	X	□	□	□
4. Were objective, standard criteria used for measurement of the condition?	X	□	□	□
5. Were confounding factors identified?	X	□	□	□
6. Were strategies to deal with confounding factors stated?	X	□	□	□
7. Were the outcomes measured in a valid and reliable way?	X	□	□	□
8. Was appropriate statistical analysis used?	X	□	□	□

Overall appraisal: Include **(X)** Exclude ( ) Seek further info ( )
Score: 100%
Comments: Not applicable

JBI Critical Appraisal Checklist for Analytical Cross Sectional Studies
Reviewer: RLAN
Date: March, 13, 2023
Authors: Bahrami A, Mohebali M, Reisi Nafchi H, Raoofian R, Kazemirad E, Hajjaran H.
Year: 2022
Record number: https://pubmed.ncbi.nlm.nih.gov/36694571/

	Yes	No	Unclear	Not applicable
1. Were the criteria for inclusion in the sample clearly defined?	X	□	□	□
2. Were the study subjects and the setting described in detail?	X	□	□	□
3. Was the exposure measured in a valid and reliable way?	X	□	□	□
4. Were objective, standard criteria used for measurement of the condition?	X	□	□	□
5. Were confounding factors identified?	X	□	□	□
6. Were strategies to deal with confounding factors stated?	X	□	□	□
7. Were the outcomes measured in a valid and reliable way?	X	□	□	□
8. Was appropriate statistical analysis used?	X	□	□	□

Overall appraisal: Include **(X)** Exclude ( ) Seek further info ( )
Score: 100%
Comments: Not applicable
